# Hemodynamic impact of chest compression location during cardiopulmonary resuscitation guided by transesophageal echocardiography

**DOI:** 10.1186/s13054-023-04575-7

**Published:** 2023-08-19

**Authors:** Felipe Teran, Clark G. Owyang, Manuel Martin-Flores, Derek Lao, Andrea King, Joanna Palasz, Joaquin D. Araos

**Affiliations:** 1 Department of Emergency Medicine, Weill Cornell Medicine/New York Presbyterian Hospital, 525 East 68th St, NY 10065 New York, USA; 2 Division of Pulmonary and Critical Care Medicine, Weill Cornell Medicine/New York Presbyterian Hospital, New York, USA; 3 Department of Clinical Sciences, College of Veterinary Medicine, Cornell University, Ithaca, NY USA; 4 College of Agricultural and Life Sciences, Cornell University, Ithaca, NY USA

## Dear Editor,

Sudden cardiac arrest (CA) continues to be a significant public health problem, acting as a primary contributor to both disease and death worldwide. Current guidelines for cardiopulmonary resuscitation (CPR) during CA define the lower half of the sternum as the surface landmark for standard chest compression (CC). External CC is believed to facilitate blood flow by augmenting intrathoracic pressure and/or exerting direct compression on the left ventricle (LV) [1].

However, recent studies have scrutinized the relevance of CC location suggesting that specific hand position on the chest may influence CPR effectiveness [2]. Importantly, standard CC positioning may paradoxically impede forward flow in many patients; in over 50% of adults, the standard CC position is over the left ventricular outflow tract (LVOT) and aortic root [3]. Supporting this theory, published work in a cohort of out-of-hospital cardiac arrest patients by our team has shown that obstruction of the LVOT is common and associated with worse survival outcomes [4].

Recently, Marshall et al. [5], reported a swine model of CA where transthoracic echocardiography (TTE) was used to locate thoracic landmarks corresponding to (1) the area of standard CC, defined as the sternal midline, at the level of the aortic root, and (2) the intersection of the parasternal long and short axis of the LV which was defined as the LV CC site. Indices of forward flow, such as cardiac output, blood pressure, and cerebral perfusion, improved when compressions were performed at the level of the LV rather than the LVOT (i.e., standard CC). These data strongly support that CC location is paramount to CPR hemodynamic optimization and that inadequate CC location may partially or totally obstruct the LVOT.

**Fig. 1:**
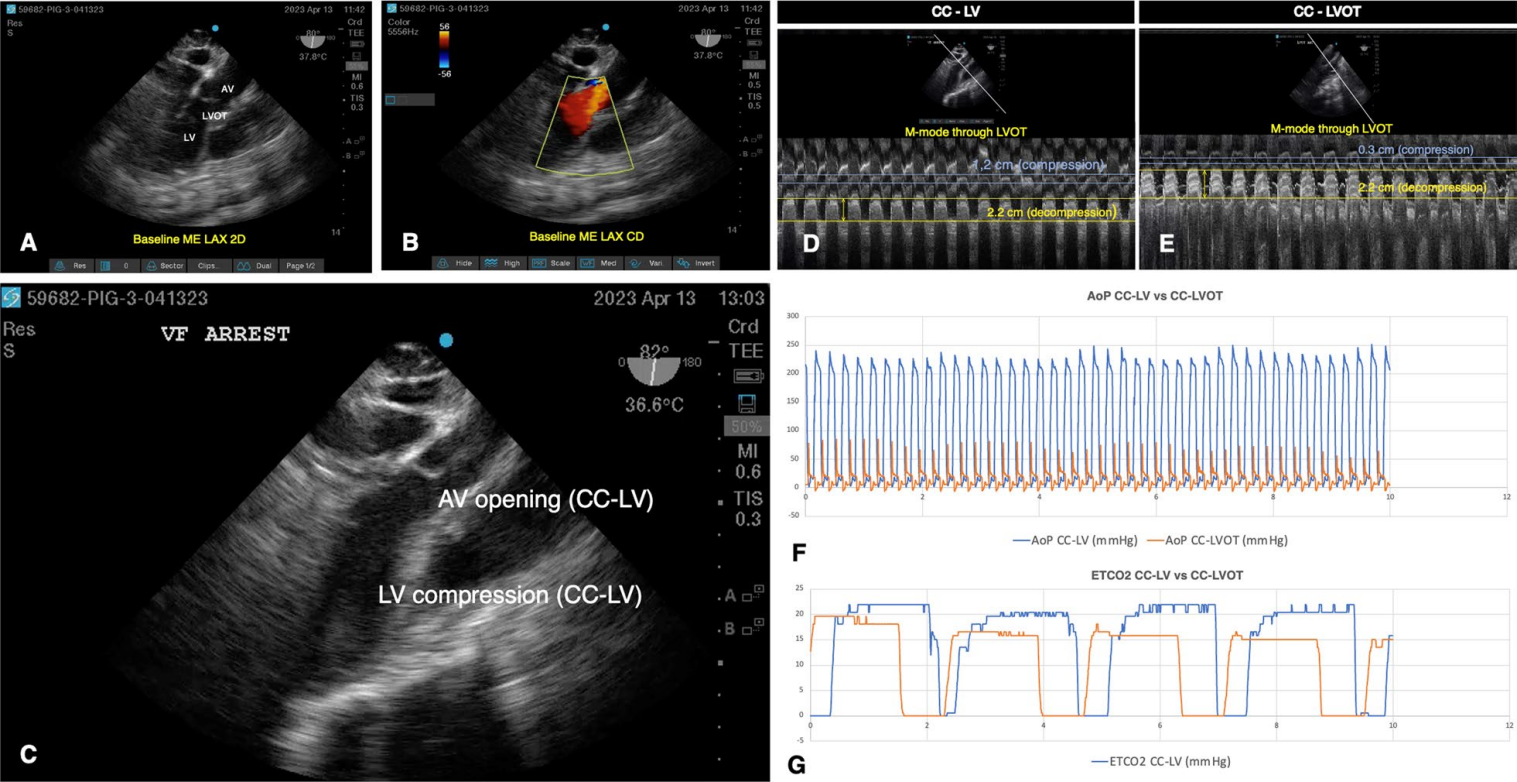
Summary of echocardiography and hemodynamic data. **A** TEE 2D image of midesophageal long axis view (MELAX) during baseline period depicting anatomic structures of interest: the left ventricle (LV), the left ventricular outflow tract (LVOT) and the aortic valve (AV). **B** MELAX view depicting Color Doppler (CD) through the LVOT. Red color in the Doppler scale corresponds to direction of blood flow from the LV, through the LVOT, and into the ascending aorta. **C** Shows MELAX image of mid-compression cycle when CCs were being directed to the LV (CC-LV). Anteroseptal wall of the LV is being compressed against the inferolateral wall of the LV and the AV is beginning to open. **D** and **E** M-mode image through the LVOT during CC-LV (**D**) and CC-LVOT (**E**) with unidimensional measurements of the diameter of this structure during the compression (yellow lines) and decompression (blue lines) phases of CPR. Overlapping hemodynamic measurements corresponding to simultaneous 10 s epochs comparing the two CC locations are shown in **F** (aortic pressures) and **G** (ETCO_2_)

To our knowledge, no previous report exists which simultaneously quantifies the patency of the LVOT with relevant CPR hemodynamics. Using a swine model of ventricular fibrillation (VF)-induced CA as part of the preparation for a prospective study of a larger sample size, we studied the hemodynamic effects of mid-LV (CC-LV) and LVOT chest compressions (CC-LVOT) in one pig, with continuous transesophageal echocardiography (TEE) imaging during CPR. After IACUC approval, an anesthetized pig was mechanically ventilated with tidal volume = 10 mL/kg, RR = 15 breaths/min, PEEP = 5 cmH_2_O. The middle port of a triple lumen pulmonary artery catheter (Swan-Ganz Thermodilution Pace port Catheter, 7.5 F, 110 cm, Edwards Lifesciences) was used to advance a pacing electrode into the RV. Ascending aortic pressures (AoP) were monitored using a 5Fr micromanometer catheter (Millar, ADInstruments) that was advanced through an introducer sheath located in the femoral artery. Proper catheter placement was confirmed with ultrasound. Following baseline instrumentation, the thoracic locations corresponding to the mid-LV and LVOT were confirmed and marked using TTE to localize the aortic valve (AV), papillary muscles and apex of the LV in two orthogonal planes. A TEE probe (TEExi/8-3 MHz Transducer, Fujifilm SonoSite) was then used to obtain a mid-esophageal long axis view (ME LAX) for real-time evaluation of the LVOT during CPR (Fig. 1A, B). A pacing probe (Chandler Transluminal V-Pacing probe, 2.4 F, 135 cm, Edwards Lifesciences) was advanced at this point to induce VF. CPR was performed with a piston-driven mechanical compression device (Life-Stat® 1008, Michigan Instruments, Grand Rapids, MI) at rate of 100 compressions/min, along with manual ventilations at a 30:2 ratio. Under real-time TEE guidance, CPR was delivered alternating between CC-LV and CC-LVOT in one-minute intervals. The rate and depth of CC was unchanged.

During the CC-LVOT interval phase, we observed near complete closure of the LVOT with concomitant lower AoP and ETCO_2_. Figure 1 C shows TEE image example of CC-LV where the LV is being targeted, leading to opening of the AV, and representing the “ideal” CC location. Images **D** and **E** are M-mode images through the LVOT generated from video clips corresponding to each CC location interval. Overlapping aortic pressures (**F**) and end-ETCO_2_ (**G**) of simultaneous 10 s epochs comparing the two CC locations are presented. During intervals of CC-LV we consistently observed higher AoP and end-tidal CO_2_ (ETCO_2_)_._ During compressions directed over the LV, we observed patency of the AV and Color Doppler (CD) reflecting flow through the LVOT (Fig. 1F, G).

Our observations and these preliminary data suggest that CC location is a key factor in determining effective generation of forward flow during CPR. TEE-facilitated CPR can show partial or complete obstruction of the LVOT during CC leading to sub-optimal hemodynamics and resuscitation. Further studies are needed to characterize the magnitude of these hemodynamic changes during TEE-guided CPR and the implications for clinical outcomes.

## Data Availability

The datasets used and/or analyzed during the study are available from the corresponding author on reasonable request.
